# Sulfide promotes tolerance to drought through protein persulfidation in Arabidopsis

**DOI:** 10.1093/jxb/erad165

**Published:** 2023-05-06

**Authors:** Ana Jurado-Flores, Angeles Aroca, Luis C Romero, Cecilia Gotor

**Affiliations:** Instituto de Bioquímica Vegetal y Fotosíntesis, Consejo Superior de Investigaciones Científicas and Universidad de Sevilla, Avenida Américo Vespucio, 49, 41092 Sevilla, Spain; Instituto de Bioquímica Vegetal y Fotosíntesis, Consejo Superior de Investigaciones Científicas and Universidad de Sevilla, Avenida Américo Vespucio, 49, 41092 Sevilla, Spain; Instituto de Bioquímica Vegetal y Fotosíntesis, Consejo Superior de Investigaciones Científicas and Universidad de Sevilla, Avenida Américo Vespucio, 49, 41092 Sevilla, Spain; Instituto de Bioquímica Vegetal y Fotosíntesis, Consejo Superior de Investigaciones Científicas and Universidad de Sevilla, Avenida Américo Vespucio, 49, 41092 Sevilla, Spain; University of Cologne, Germany

**Keywords:** Abiotic stress, abscisic acid, amino acids, Arabidopsis, comparative proteomics, dimedone switch, drought, persulfidation, reactive oxygen species

## Abstract

Hydrogen sulfide (H_2_S) is a signaling molecule that regulates essential plant processes. In this study, the role of H_2_S during drought was analysed, focusing on the underlying mechanism. Pretreatments with H_2_S before imposing drought on plants substantially improved the characteristic stressed phenotypes under drought and decreased the levels of typical biochemical stress markers such as anthocyanin, proline, and hydrogen peroxide. H_2_S also regulated drought-responsive genes and amino acid metabolism, and repressed drought-induced bulk autophagy and protein ubiquitination, demonstrating the protective effects of H_2_S pretreatment. Quantitative proteomic analysis identified 887 significantly different persulfidated proteins between control and drought stress plants. Bioinformatic analyses of the proteins more persulfidated in drought revealed that the most enriched biological processes were cellular response to oxidative stress and hydrogen peroxide catabolism. Protein degradation, abiotic stress responses, and the phenylpropanoid pathway were also highlighted, suggesting the importance of persulfidation in coping with drought-induced stress. Our findings emphasize the role of H_2_S as a promoter of enhanced tolerance to drought, enabling plants to respond more rapidly and efficiently. Furthermore, the main role of protein persulfidation in alleviating reactive oxygen species accumulation and balancing redox homeostasis under drought stress is highlighted.

## Teaser

Sulfide promotes enhanced tolerance to drought through the persulfidation of proteins mainly involved in the balance of redox homeostasis, allowing plants to respond more rapidly and efficiently after exposure to drought.

## Introduction

Drought is one of the most important abiotic stress conditions in plants and adversely affects plant growth and crop production worldwide (Xiong *et al.*, 2002). Plants have the capacity to respond to drought stress by using multiple strategies including morphological changes, biochemical responses, and physiological adaptations (Hura *et al.*, 2022). Dehydration stimulates the production of the phytohormone abscisic acid (ABA) and induces stomatal closure to reduce water loss (Leckie *et al.*, 1998). This stomatal closure also reduces CO_2_ uptake and hence alters photosynthesis, reducing plant growth and yield (Davies, 2006). In addition, the synthesis of the osmolyte proline is activated under water deprivation. Another consequence of drought stress is an increase in reactive oxygen species (ROS) production in the cellular compartments, with consequent activation of the antioxidant defense system, including ROS-scavenging enzymes such as superoxide dismutase, ascorbate peroxidase, catalase, glutathione peroxidase, and peroxiredoxin, and the induction of antioxidant molecules, such as ascorbic acid and reduced glutathione (Reddy *et al.*, 2004; Cruz de Carvalho, 2008; Xu *et al.*, 2017). Therefore, there is increasing evidence of the importance of ROS regulation in abiotic stress responses of plants.

Hydrogen sulfide (H_2_S) has historically been considered toxic for any living form, but is currently demonstrated as an important signaling molecule along with nitric oxide, carbon monoxide, and hydrogen peroxide (H_2_O_2_) that regulates essential processes in both mammals and plants (Kimura, 2002; Aroca *et al.*, 2020). H_2_S has been shown to be involved in a wide range of biological processes in plants, including various developmental and physiological aspects, but also stress responses that cope with heavy metal toxicity, salinity, and heat, among others (Zhang *et al.*, 2015; Zhao *et al.*, 2018; Zhou *et al.*, 2018; Corpas, 2019; Gotor *et al.*, 2019). H_2_S alleviates the negative effects of certain abiotic stresses such as drought stress, which is mitigated by sulfide through the regulation of ABA-dependent stomatal movement (Jin *et al.*, 2011; Papanatsiou *et al.*, 2015; Aroca *et al.*, 2021b). Besides, the induction of autophagy is among the different responses of plant adaptation to abiotic stress (Avin-Wittenberg, 2019; Signorelli *et al.*, 2019), and substantial findings have highlighted the role of H_2_S in negatively regulating the autophagy induced under stress conditions (Gotor *et al.*, 2013, 2022).

However, the mechanism of action of H_2_S is still unclear but must relate to its chemical properties. Thus, H_2_S shows affinity for metal centers of metalloproteins such as cytochrome *c* oxidase (Filipovic *et al.*, 2018; Vitvitsky *et al.*, 2018) and can also react with other small reactive oxygen and nitrogen species (Li and Lancaster, 2013). A third mechanism of action of sulfide that has been studied in more depth is that the thiol group (-SH) of cysteine residues can be modified by H_2_S into a persulfide group (-SSH) that affects protein stability, structure, function, activity, or localization within cells (Aroca *et al.*, 2015). This oxidative post-translational modification of cysteine residues caused by H_2_S is called persulfidation and has been intensively studied in the last decade in mammalian and plant systems (Mustafa *et al.*, 2009; Aroca *et al.*, 2015).

In plants, several recent proteomic analyses of label-persulfidated proteins have shown that almost 13% of the entire annotated proteome of Arabidopsis was a target for persulfidation (Aroca *et al.*, 2015, 2017, 2018; Jurado-Flores *et al.*, 2021, 2023). Furthermore, a detailed analysis has revealed that the identified persulfidated proteins are involved in a wide range of important biological processes, such as carbon metabolism, plant growth and development, plant response to stresses, RNA translation, and protein degradation (Aroca *et al.*, 2017). In addition, ABA-regulated stomatal movement has been demonstrated to be regulated by H_2_S-mediated persulfidation of different components of the ABA signaling pathway, such as open stomata 1 (OST1)/SNF1-RELATED PROTEIN KINASE 2.6 (SnRK2.6) (Chen *et al.*, 2020) and NADPH oxidase respiratory burst oxidase homolog protein D (RBOHD) (Shen *et al.*, 2020). More specifically, persulfidation has been demonstrated as the molecular mechanism through which sulfide regulates autophagy in plants (Gotor *et al.*, 2022). Thus, persulfidation of the autophagy-related 4A cysteine protease (ATG4a) has been reported as the mechanism of action of H_2_S in the regulation of the ABA-induced autophagic process (Laureano-Marín *et al.*, 2020). In a similar way, persulfidation of autophagy-related protein 18a (ATG18a) regulates the selective degradation of the endoplasmic reticulum through autophagy (Aroca *et al.*, 2021a). Autophagy is an important plant response to drought stress, as it is a recycling pathway that removes damaged components, proteins, and organelles affected by ROS under stress conditions to reset the cell status. Therefore, a deep study of the regulation of autophagy by H_2_S might help in developing future approaches to conferring plant tolerance to drought to cope with climatic change.

In the present work, we studied the protective role of endogenous H_2_S during drought stress focusing on the molecular mechanism of protein persulfidation by which H_2_S exercises its action. In addition, we analysed the effect of exogenous H_2_S pretreatment by studying the changes of biochemical parameters and the molecular pathways of protein degradation activated to survive drought stress.

## Materials and methods

### Plant material and treatments

**Fig. 1.**
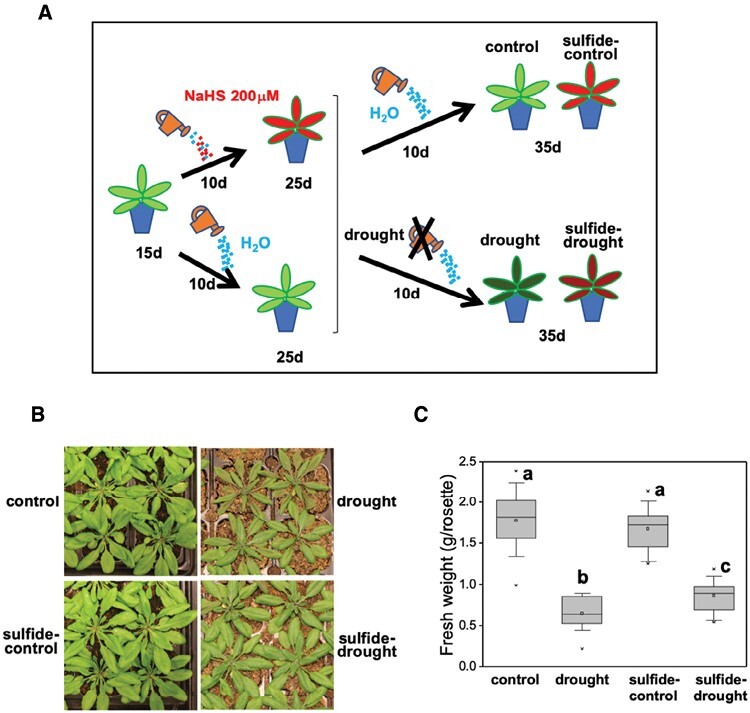
Effect of NaHS pretreatment on the phenotype of Arabidopsis plants under drought stress. (A) Scheme of the experiment performed. Fifteen-day-old plants grown in soil under physiological conditions were divided into two batches, one batch irrigated with water and the other with 200 μM NaHS for 10 additional days. Each batch was subsequently divided into two new batches and subjected to water irrigation or drought for 10 additional days. At the end of the full treatment, four different plant samples were obtained and named as shown in the figure (control, sulfide–control, drought, and sulfide–drought). (B) Phenotypes of the different plant samples at the end of the full treatment. (C) Quantification of the plant fresh weight. Values are means ±SD (*n*=3). Different letters indicate statistically significant differences (ANOVA, Fisher’s least significant difference test, *P*<0.05).

Arabidopsis wild type and a line expressing green fluorescent protein (GFP)–ATG8a under the control of the Arabidopsis *UBQ10* promoter (Nottingham Arabidopsis Stock Centre) were used in this work. Plant growth conditions were 16 h of light (140 μmol m^−2^ s^−1^) at 22 °C and 8 h of dark at 20 °C. For exogenous sulfide treatment, the established sulfide donor NaHS was used, which generates neutral H_2_S and the anionic forms HS^−^ and S^2−^ in solution (Kabil and Banerjee, 2010). Thus, 15-day-old plants grown in soil were divided into two batches, and one batch was irrigated with water and the other with 200 μM NaHS for 10 additional days. After this period, each batch was subsequently divided into two new batches and subjected to water irrigation or drought for another 10 additional days as described in Fig. 1A.

### Determination of anthocyanin, hydrogen peroxide, and thiol compound contents

For the quantification of the anthocyanin content, approximately 40–70 mg of leaf tissue was homogenized in 1 ml of propanol:HCl:water (18:1:81) and further extracted in a boiling-water bath for 3 min. The mixture was centrifuged at 5000 *g* for 40 min. The absorbance of the supernatant was measured at 535 and 650 nm, and referred to the fresh weight as described (Laureano-Marín *et al.*, 2016).

To quantify hydrogen peroxide, 50 mg of leaf tissue was ground in liquid nitrogen with 250 μl of 50 mM phosphate buffer, pH 7.4, vortexed and shaken continuously at room temperature for 30 min. Samples were centrifuged at 4 °C at 12 000 *g* for 10 min. Fluorescence quantification of H_2_O_2_ was performed in the supernatant after incubation with Amplex Red reagent (Thermo Fisher Scientific) and horseradish peroxidase using 560 nm excitation and 590 nm emission filters. Standard calibration curves were obtained with known H_2_O_2_ concentrations.

To quantify the total Cys and glutathione contents, thiols were extracted, reduced with NaBH_4_, and quantified by reverse-phase HPLC after derivatization with monobromobimane (Thermo Fisher Scientific) as described previously (Dominguez-Solis *et al.*, 2001).

### Quantification of endogenous H_2_S

Sulfide quantification was performed by ultra-performance liquid chromatography–tandem mass spectrometry (UPLC-MS/MS) using 100 mg of leaf tissue as previously described using monobromobimane derivatization (Tan *et al.*, 2017). The concentrations were calculated based on a standard curve of NaHS concentrations from 2.5 to 100 µM.

### Amino acid determination

Free amino acid quantification was performed by UPLC-MS/MS. Approximately 100 mg of frozen leaf tissue was homogenized in 1.5-ml Eppendorf tubes for 2 min at maximum speed with a Retsch ball mill (MM400, Retsch). The metabolites were extracted from each aliquot in 0.4 ml of 0.1 M HCl and 0.1% formic acid with shaking for 30 min at 4 °C in an Eppendorf ThermoMixer C. Samples were centrifuged for 15 min at 4700 *g* at 4 °C.

UPLC separation of the amino acids was performed using the ExionLC UPLC system (Sciex) with a reversed-phase column (100 mm×4.6 mm×100 Å particle, Kinetex XB-C18). The mobile phases were 0.1% formic acid in H_2_O (Buffer A, HPLC/LCMS grade) and 0.1% formic acid in acetonitrile (Buffer B, HPLC/LC-MS grade). A 5 µl sample was loaded per injection, and the gradient, applied at a flow rate of 600 µl min^−1^, was as follows: 3 min 100% A, 3 min linear gradient from 100% A to 80% A, 2 min linear gradient from 80% A to 50% A, 1 min hold at 50% A, 1 min linear gradient from 50% to 100% A, and hold at 100% A to re-equilibrate the column for 4 min (14 min total run time).

Mass spectra were acquired using a QTRAP 6500+ triple quadrupole (Sciex) equipped with an electrospray ionization source operating in the positive ionization mode using an ion spray voltage of 4500 V. The other ESI parameters were as follows: curtain gas, 35 psi; collision gas, medium; temperature, 500 °C; nebulizer gas (GS1), 60 psi; and heater gas (GS2), 60 psi. Data were acquired with Analyst 1.7 software in the multiple reaction monitoring (MRM) mode with a detection window of 60 s. The ionization adducts measured [M+H^+^], and optimized declustering potential (DP) and collision energy (CE) for each MRM transition were: Cys: Q1 122.0 Da, Q3 76.0 Da, DP 40.0 V, CE 17.0 V; Met: Q1 150,1 Da, Q3 104.0 Da, DP 6.0 V, CE 15.0 V; Gly: Q1 76.0 Da, Q3 30.0 Da, DP 6.0 V, CE 19.0 V; Ala: Q1 90.0 Da, Q3 44.0 Da, DP 6.0 V, CE 17.0 V; Ser: Q1 106.0 Da, Q3 60.0 Da, DP 6.0 V, CE 15.0 V; Pro: Q1 116.0 Da, Q3 70.0 Da, DP 20.0 V, CE 21.0 V; Val: Q1 118.0 Da, Q3 55.0 Da, DP 11.0 V, CE 27.0 V; Thr: Q1 120.0 Da, Q3 103.0 Da, DP 105.0 V, CE 25.0 V; Ile: Q1 132.0 Da, Q3 86.0 Da, DP 8.0 V, CE 13.0 V; Leu: Q1 132.0 Da, Q3 86.0 Da, DP 8.0 V, CE 1.0 V; Asp: Q1 134.0 Da, Q3 74.0 Da, DP 7.0 V, CE 19.0 V; Lys: Q1 147.0 Da, Q3 84.0 Da, DP 15.0 V, CE 23.0 V; Glu: Q1 148.0 Da, Q3 84.0 Da, DP 21.0 V, CE 21.0 V; His: Q1 156.0 Da, Q3 110.0 Da, DP 16.0 V, CE 19.0 V; Phe: Q1 166.0 Da, Q3 103.0 Da, DP 11.0 V, CE 37.0 V; Arg: Q1 175.0 Da, Q3 70.0 Da, DP 40.0 V, CE 27.0 V; Tyr: Q1 182.0 Da, Q3 165.0 Da, DP 20.0 V, CE 13.0 V; Gln: Q1 146.9 Da, Q3 84.1 Da, DP 16.0 V, CE 23.0 V; Trp: Q1 204.9 Da, Q3 145.9 Da, DP 6.0 V, CE 23.0 V; and Asn: Q1 132.9 Da, Q3 86.9 Da, DP 6.0 V, CE 13.0 V. Data were processed with Sciex OS software for peak integration and quantification.

### Immnunoblot analyses

Plant leaf material (300 mg) was ground in liquid nitrogen using a mortar and pestle with 450 μl of extraction buffer (100 mM Tris–HCl, pH 7.5, 400 mM sucrose, 1 mM EDTA, 10 mg ml^−1^ sodium deoxycholate, 0.1 mM phenylmethylsulfonyl fluoride, 10 mg ml^−1^ pepstatin A and 4% (v/v) protease inhibitor cocktail (Roche)) and was centrifuged at 500 *g* for 10 min to obtain the supernatant fraction. The total amount of protein in the resulting supernatant was determined using a previously described method (Bradford, 1976). Leaf protein extracts were subjected to an immunoblot analysis using SDS-PAGE on 10% or 15% (w/v) polyacrylamide gels before being transferred to a polyvinylidene fluoride membrane (Bio-Rad) according to the manufacturer’s instructions. For immunoblot analyses, the antibodies anti-GFP (Bioscience), anti-ATG8 (Agrisera), anti-UBQ (Santa Cruz Biotechnology), and secondary antibodies were diluted 1:1000, 1:5000, 1:1000, and 1:30 000, respectively, in phosphate-buffered saline (PBS) containing 0.1% Tween 20 (Sigma-Aldrich) and 5% milk powder. The ECL Select Western blotting Detection Reaction (GE Healthcare) was used to detect proteins with horseradish peroxidase-conjugated anti-rabbit secondary antibodies. For a protein loading control, the membrane before immunodetection was stained with Ponceau S (Sigma-Aldrich) to detect all protein bands.

### Real-time RT-PCR

Quantitative real-time RT-PCR was used to analyse the expression of several drought-marker genes and the ATG8 gene family. Total RNA was extracted from Arabidopsis leaves using Qiagen RNeasy Plant Mini Kit. RNA was reverse transcribed using oligo (dT) and the QuantiTect Reverse Transcription Kit (Qiagen) according to the manufacturer’s instructions. Gene-specific primers for each gene were designed using Vector NTI Advance 10 software (Supplementary Table S1). Real-time PCR was performed using AceQ SYBR qPCR Master Mix (Vazyme), and the signals were detected on an iCYCLER (Bio-Rad) according to the manufacturer’s instructions. The cycling profile consisted of 95 °C for 10 min followed by 45 cycles of 95 °C for 15 s and 60 °C for 1 min. The expression levels of the genes of interest were normalized to the constitutive *UBQ10* gene by subtracting the cycle threshold value of *UBQ10* from the cycle threshold value of the gene. The results are shown as means ±SD for at least three independent RNA samples.

### Dimedone-switch method and proteomics

The protocol was performed as previously described (Aroca *et al*., 2022). Briefly, Arabidopsis leaves were ground to a fine powder in liquid nitrogen, resuspended in cold PBS lysis buffer (PBS 1× pH 7.4, 2% SDS, and 1 mM EDTA), together with 5 mM 4-chloro-7-nitrobenzofurazan (Cl-NBF, Sigma-Aldrich) and 1% protease inhibitor, incubated at 37 °C for 30 min, protect from light. A methanol/chloroform precipitation was performed, and protein pellets were washed with cold methanol and dried. The dried proteins were dissolved in 50 mM PBS with 2% SDS, incubated with 100 µM DCP-Bio1 (Kerafast) at 37 °C for 1.5 h, precipitated and finally dissolved in 50 mM PBS with 0.1% SDS. Proteins were incubated with Sera-Mag Magnetic Streptavidin beads (Cytiva) at 4 °C overnight with agitation. The microtubes containing the magnetic beads were located in a magnet and the beads separated from the supernatant washed with PBS 1× supplemented with 0.001% Tween-20 several times. After washing, the beads were incubated with 2.25 M ammonium hydroxide overnight at room temperature, the final supernatants transferred to fresh microtubes, and the beads discarded. Samples were then neutralized with formic acid, and protein concentration was determined. A total of 50 µg of proteins was trypsinized and analysed by LC-MS/MS. Peptide identification was performed using PEAKS Studio (BSI, Canada). The search settings were: precursor Δ*m* tolerance=10 ppm, fragment Δ*m* tolerance=0.2 Da, missed cleavages=2, modifications of lysine: NBF (mass shift: 163.0012), and modifications of cysteines hydrolysed DCP-Bio1 (mass shift: 168.0786) or NBF (mass shift: 163.0012). The mass spectrometry proteomic data have been deposited to the ProteomeXchange Consortium via the PRIDE (Vizcaino *et al.*, 2016) partner repository with the identifier PXD039999.

Protein functional analysis and classification were performed with MapMan (Thimm *et al.*, 2004). The functional enrichment analysis and functional annotation of gene lists were performed using the web server DAVID (Dennis *et al.*, 2003; Sherman *et al.*, 2022).

## Results

### H_2_S treatment promotes drought tolerance in Arabidopsis

To gain a deeper understanding of the mechanism of H_2_S action regulating plant adaptation to drought stress, we established an experimental system of plants subjected to drought under different irrigation regimes. Wild-type Arabidopsis plants expressing the GFP–ATG8a fusion protein under the control of the *UBQ10* promoter were grown for 15 d in soil and divided into two batches, one batch water-irrigated and the other treated with 200 µM NaHS for 10 additional days. After this period, each batch was subsequently divided into two and subjected to water irrigation or drought for 10 additional days. After the whole treatment, we had four different plant samples that were named control, drought, sulfide–control, and sulfide–drought (Fig. 1A). Plants subjected to drought showed the characteristic phenotypes of wilting and browning leaves along with a considerable reduction in fresh weight. However, sulfide treatment imposed before drought stress substantially improved the phenotypic traits of plants (Fig. 1B, C).

**Fig. 2.**
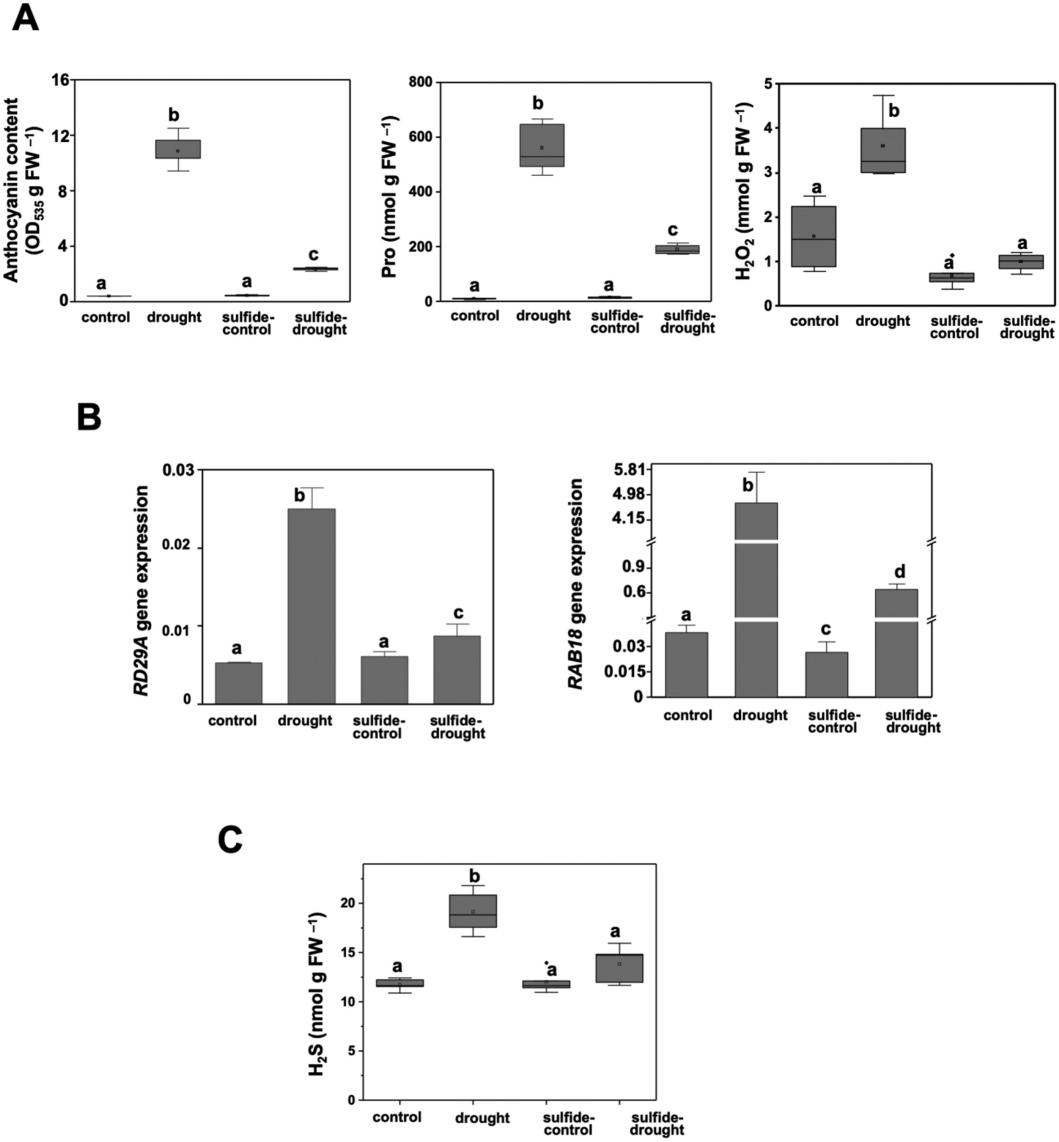
Hydrogen sulfide effects on biochemical and transcriptional parameters. (A) Determination of biochemical markers. Anthocyanin, proline and H_2_O_2_ contents per fresh weight were measured in plant samples treated as indicated. (B) Drought-response gene expression. Real-time RT-PCR analysis of *RD29A* (At5g52310) and *RAB18* (At5g66400) was performed in the plant samples treated as indicated. The transcript levels were normalized to the constitutive *UBQ10* (At4g05320) gene. (C) Quantification of endogenous H_2_S in plant samples treated as indicated. Values are means ±SD (*n*=3). Different letters indicate statistically significant differences (ANOVA, Fisher’s least significant difference test, *P*<0.05).

To further corroborate the beneficial effect of sulfide pretreatments in plants subjected to drought stress, several analyses were performed, including quantification of different biochemical parameters that are typical markers in plants affected by a stress, such as the content of anthocyanin, proline, and H_2_O_2_ (Fig. 2A). Anthocyanin content was more than 25-fold higher in plants under drought than in control or sulfide–control plants, which is indicative of plants suffering drought stress, while in sulfide-pretreated plants subjected to drought, the anthocyanin content was significantly (*P*<0.05) reduced. Similarly, the effect of sulfide pretreatment was observed when proline content was determined, which showed a huge 50-fold increase after drought stress compared with control or sulfide–control plants. The level of proline was considerably reduced in sulfide-pretreated plants under drought compared with plants without pretreatment under the same drought conditions. The substantial reduction of both parameters in sulfide-pretreated plants clearly demonstrates an improvement by sulfide in plant tolerance (Fig. 2A).

When plants are exposed to adverse environmental conditions, cell homeostasis is altered, causing ROS overproduction and further oxidative damage. Therefore, H_2_O_2_ levels during drought stress were determined and the results showed a significant (*P*<0.05) production of H_2_O_2_ when compared with control or sulfide–control plants, as expected. Sulfide pretreatment decreased significantly (*P*<0.05) H_2_O_2_ content in plants under drought conditions, demonstrating the relief effect of sulfide in terms of ROS production (Fig. 2A).

At the molecular level, a protective effect of sulfide was also observed. The transcript levels of the drought-responsive genes *RD29A* and *RAB18* were significantly (*P*<0.05) induced under drought conditions, demonstrating that plants were affected by the stress induced by drought. However, plants pretreated with sulfide and subjected to drought showed significantly (*P*<0.05) lower levels of these two stress markers (Fig. 2B). Overall, our results demonstrate that the drought stress condition is alleviated by sulfide.

In addition, we determined the content of H_2_S in plant samples and observed a significant (*P*<0.05) induced endogenous level of H_2_S under drought. However, when plants were provided with exogenous sulfide, the endogenous level of H_2_S was reduced to concentrations similar to those of control plants even under drought conditions (Fig. 2A). This finding could suggest that plants respond to drought by increasing the available sulfide to combat the drought-induced stress, and the pretreatment with exogenous sulfide avoids this requirement.

### H_2_S treatment reverts the amino acid accumulation upon drought

**Fig. 3.**
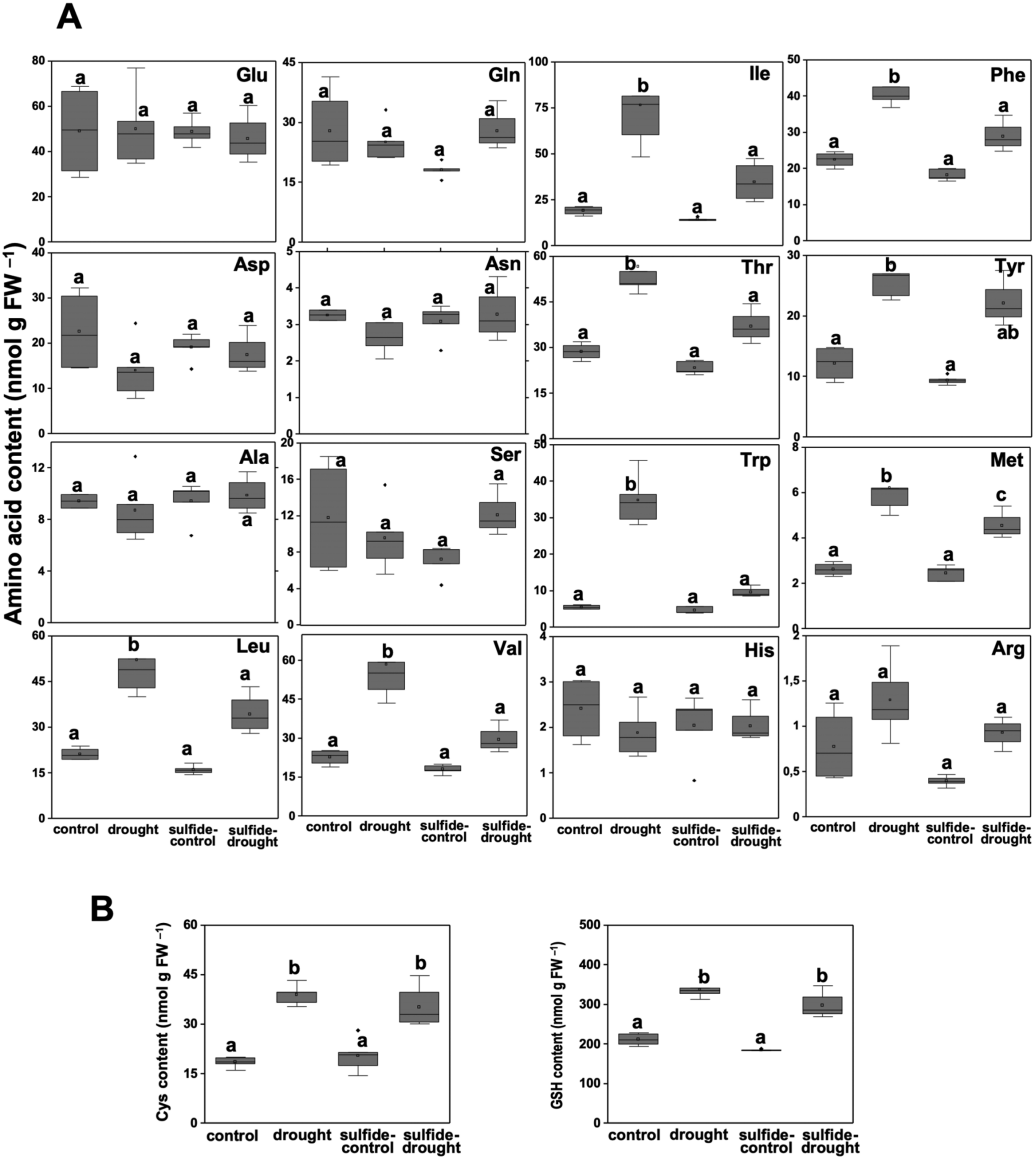
Analysis of NaHS pretreatment on content of free amino acids and thiol compounds under drought stress in plants treated as indicated. (A) Levels of individual amino acid were determined by UPLC-MS/MS. (B) Cys and glutathione contents were determined by HPLC after derivatization. Values are means ±SD (*n*=3). Different letters indicate statistically significant differences (ANOVA, Fisher’s least significant difference test, *P*<0.05).

Plant metabolism must adapt to an abiotic stress, and in doing so, changes in amino acid metabolism occur. In this stress situation, amino acids are required as precursors for the synthesis of stress-induced proteins and secondary metabolites, such as flavonoids and glucosinolates. Further, accumulation of proline is a response to drought-induced osmotic stress, as it is a major compatible osmolyte in plants (Delauney and Verma, 1993). Drought also impedes the proper functioning of photosynthesis resulting in the lack of energy and depletion of carbohydrate, and amino acids are used as alternative substrates for ATP production (Heinemann and Hildebrandt, 2021). Therefore, the steady state level of individual amino acids was quantified in plants subjected to our experimental conditions (Fig. 3A). We observed that Glu, Gln, Asp, Asn, Ala, Ser, His, and Arg were not affected by drought conditions, probably because they are very abundant amino acids and are connected to organic nitrogen storage (Hildebrandt, 2018). By contrast, the branched-chains amino acids Ile, Leu, and Val; the aromatic amino acids Phe, Tyr, and Trp; together with Thr and Met significantly (*P*<0.05) accumulated upon drought. Interestingly, the levels of these latter amino acids were substantially reduced when sulfide pretreatment was applied. These results indicate that sulfide pretreatment alters the amino acid metabolism under drought stress conditions.

Furthermore, a significant (*P*<0.05) increase in Cys and glutathione contents was observed in plants subjected to drought (Fig. 3B) that was similar in sulfide-pretreated plants. This result could indicate that the role of sulfide in alleviating drought stress might be unrelated to primary sulfur metabolism.

### H_2_S repressed the drought-induced cell content degradation pathways

The induction of degradation pathways of damaged cellular components, such as macroautophagy (referred to hereafter as autophagy) and the ubiquitin–proteasome system (UPS), is one of the main plant responses to environmental stresses. These two processes are highly related and function jointly to maintain cell homeostasis (Minina *et al.*, 2017; Su *et al.*, 2020; Clavel and Dagdas, 2021). Therefore, the effects of sulfide pretreatment on autophagy and bulk ubiquitination induced by drought were analysed.

**Fig. 4.**
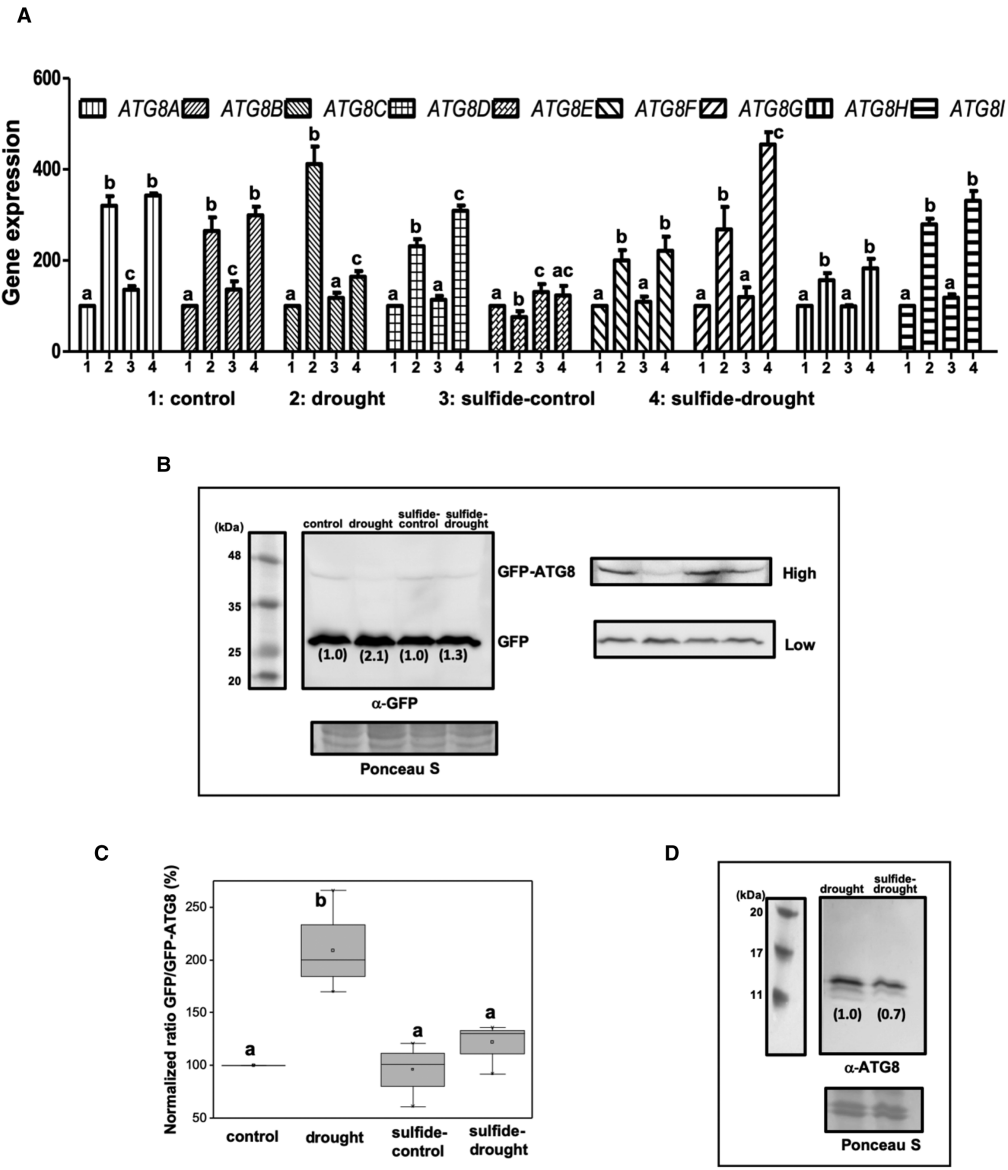
Pretreatment with NaHS regulates autophagy induced by drought stress. (A) Expression levels of the *ATG8* gene family. Real-time RT-PCR analysis of the nine Arabidopsis *ATG8* genes was performed in the plant samples obtained as shown in Fig. 1. The transcript levels were normalized to the constitutive *UBQ10* gene. Bars represent means ±SD (*n*=3). Different letters indicate statistically significant differences (ANOVA, Fisher’s least significant difference test, *P*<0.05). (B–D) Immunoblot analysis of GFP–ATG8a fusion protein using anti-GFP antibodies (B, C) and endogenous ATG8 proteins using anti-ATG8 antibodies (D). Ponceau S staining was used as the protein loading control. Quantification of the ratio free GFP/GFP–ATG8 was performed using different time exposure blots as indicated in the figure, high for GFP–ATG8 and low for free GFP. The ratio is indicated in the blots in parentheses, with a value of 1 assigned to the normalized ratio corresponding to control samples. The relative band intensities were calculated based on the control samples and normalized using the protein loading control. Values are means ±SD (*n*=4). Different letters indicate statistically significant differences (ANOVA, Fisher’s least significant difference test, *P*<0.05).

It is well established that drought stress up-regulates autophagy (Tang and Bassham, 2022), and proteins involved in autophagy have been used to monitor autophagic activity, the ATG8-family proteins being most widely used (Klionsky *et al.*, 2021). Arabidopsis contains nine different ATG8 isoforms and our results showed that the transcript levels of all the ATG8 gene family increased significantly (*P*<0.05) in plants subjected to drought. However, sulfide-pretreated plants under drought showed similar levels of up-regulation of all ATG8 transcripts, with the exception of ATG8C, which was down-regulated (Fig. 4A), suggesting that pretreatment with sulfide does not regulate autophagy at the transcriptional level. This observation is consistent with previous studies that demonstrated that sulfide regulates autophagy post-translationally through persulfidation of target proteins (Laureano-Marín *et al.*, 2020; Aroca *et al.*, 2021a; Gotor *et al.*, 2022). To analyse the progression of autophagy under our experimental conditions, total protein extracts were immunoblotted using anti-GFP antibodies to detect the fusion protein GFP–ATG8 and the free GFP in order to determine the autophagic flux. Our results showed a faint protein band corresponding to the fusion protein, because protein expression was under the control of the weak *UBQ10* promoter, and an intense protein band that corresponded to free GFP. Blots were analysed with different exposure times to visualize well the free GFP and the fusion protein, and quantify the ratio of free GFP/GFP-ATG8 for each condition (Fig. 4B). Quantification showed that autophagic flux was strongly induced under drought stress, showing a ratio double that of control plants (Fig. 4B, C). This induction was repressed by pretreating plants with sulfide, reaching levels of autophagy similar to those observed in control plants. In addition, another immunoblot analysis was performed to detect endogenous ATG8 proteins in protein extracts using anti-CrATG8 antibodies (Alvarez *et al.*, 2012), and similar results were obtained (Fig. 4D). The high level of endogenous ATG8 proteins detected under drought conditions was significantly (*P*<0.05) reduced in sulfide-pretreated plants. Therefore, our findings suggest that sulfide is also a negative regulator of drought-induced autophagy, in a similar way to that previously demonstrated for autophagy induced by nutrient deprivation, ABA treatment, or endoplasmic reticulum stress (Laureano-Marín *et al.*, 2016; Laureano-Marín *et al.*, 2020; Aroca *et al.*, 2021a).

**Fig. 5.**
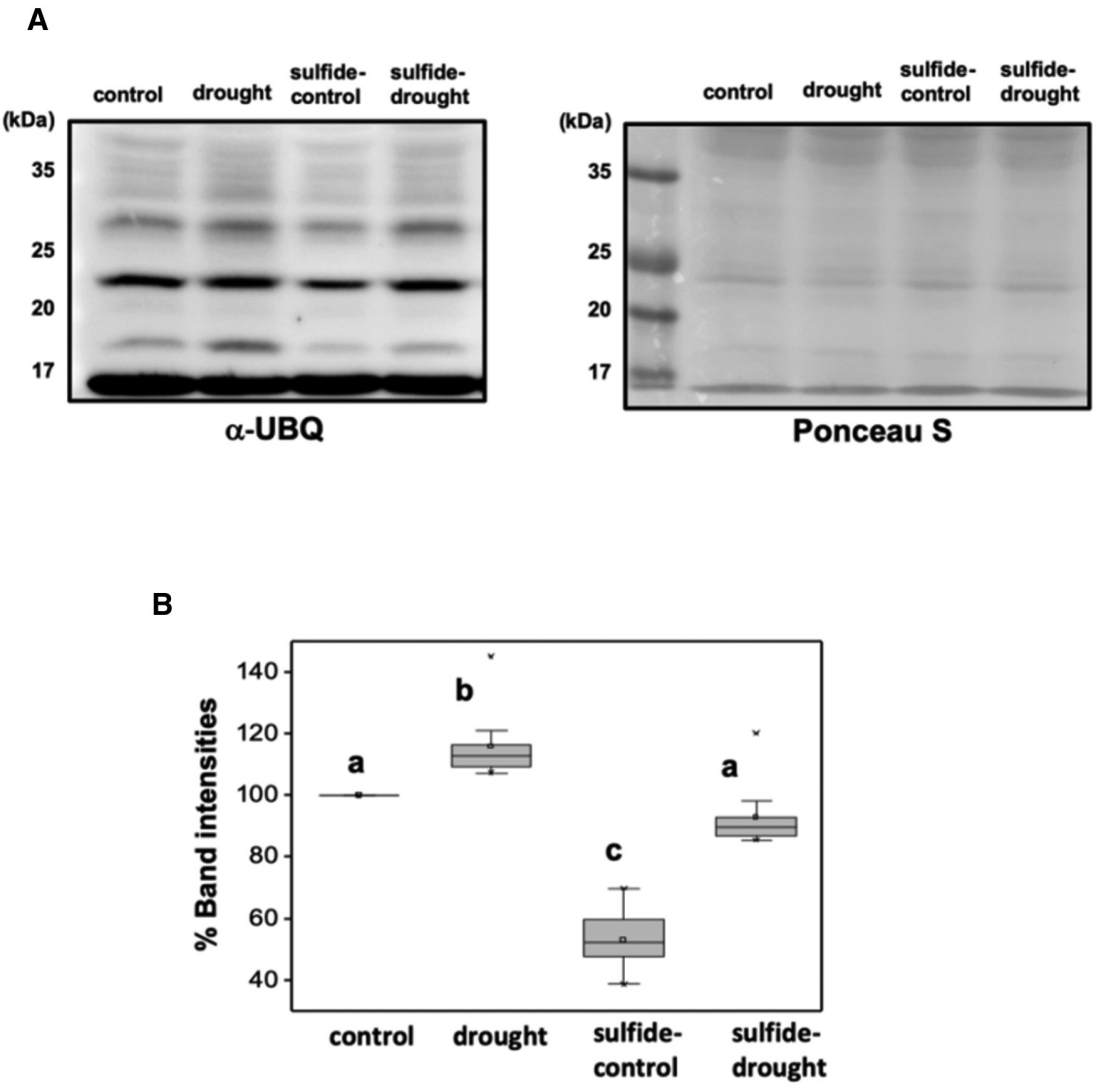
Regulation of the bulk ubiquitination by NaHS under drought stress. (A) Immunoblot analysis of ubiquitin using anti-ubiquitin antibody. Ponceau S staining was used as the protein loading control. Molecular mass is indicated on the left. (B) Relative quantification of band intensities compared with control samples and normalized with loading control. Values are means ±SD (*n*=3). Different letters indicate statistically significant differences (ANOVA, Fisher’s least significant difference test, *P*<0.05).

The UPS is another major proteolytic pathway besides autophagy. In a previous quantitative proteomic analysis for the detection of persulfidated proteins under nitrogen deprivation-induced autophagy, an over-representation of proteins related to the ubiquitin-dependent degradation pathway was identified (Jurado-Flores *et al.*, 2021). Therefore, bulk ubiquitination in plants subjected to our experimental conditions was analysed by immunoblotting using anti-UBQ antibodies (Fig. 5). Our results showed that plants subjected to drought showed a significant (*P*<0.05) increase in ubiquitinated proteins, while plants under drought stress pretreated with sulfide showed the same level of ubiquitination as control conditions (Fig. 5A, B). Therefore, sulfide pretreatment also regulated the proteolytic pathway through the UPS under drought conditions.

### Identification and quantitative comparison of persulfidated proteins between control and drought conditions

As mentioned above, the best characterized molecular mechanism of hydrogen sulfide signaling in Arabidopsis is protein persulfidation (Aroca *et al.*, 2018; Gotor *et al.*, 2019, 2022; Corpas *et al.*, 2021). In order to decipher the role of persulfidation in the promotion of drought tolerance by sulfide, a mass spectrometry-based label-free quantitative proteomic approach combined with the dimedone-switch method (Zivanovic *et al.*, 2019; Aroca *et al*., 2022) was performed in leaf tissues from 35-day-old Arabidopsis plants growing under control or under drought conditions as previously described (Fig. 1A). Protein samples from three biological replicates (independent pools) were isolated and subjected to the dimedone-switch procedure. The proteins eluted from the streptavidin beads were digested, and the peptide solutions were analysed by LC-MS/MS. A total of 2568 persulfidated proteins were identified in all samples (Supplementary Dataset S1), which is a similar amount to the previous proteins detected in Arabidopsis leaf tissues under non-stressed conditions (Aroca *et al.*, 2017). Label-free quantification revealed that 887 proteins (Supplementary Dataset S2) exhibited persulfidation levels significantly (*P*<0.05) different between both conditions, with 437 proteins more persulfidated under drought stress (Supplementary Table S2) and 450 proteins more persulfidated in control samples (Supplementary Table S3). Among the proteins that were found more persulfidated under drought, the ones with the highest level (a 24.7-fold change) were a malonyl-CoA:anthocyanidin 5-*O*-glucoside-6ʹʹ-*O*-malonyltransferase and a leucoanthocyanidin dioxygenase (13.4-fold change), which are involved in the anthocyanin-containing compound biosynthetic process. In contrast, the protein that was found to be least persulfidated under drought was the AT2S2 seed storage albumin protein.

To uncover specific biological functions and pathways involved in H_2_S signaling of drought responses, we first analysed the 437 proteins with higher level of persulfidation under drought using Database for Annotation, Visualization, and Integrated Discovery (DAVID) bioinformatic resources (*P*<0.05). Functional characterization and enrichment analysis of these proteins showed that the at least 15 Gene Ontology (GO) biological processes identified included more than 10 persulfidated proteins and were related to the observed effects of H_2_S pretreatment on drought tolerance (Table 1). The protein group with the highest fold-enrichment value corresponded to the term ‘cellular response to oxidative stress’ with 11 proteins, comprising six peroxidases, including cytosolic ascorbate peroxidase 1 (APX1), previously shown to be regulated by persulfidation of Cys32 (Aroca *et al.*, 2015, 2021b). Two copper/zinc superoxide dismutases with high persulfidation level under drought (Supplementary Table S2), two thioredoxin superfamily proteins, and a peptidemethionine sulfoxide reductase were also included. The second most enriched term was the ‘hydrogen peroxide catabolic process’, containing most of the proteins included in the term ‘cellular response to oxidative stress’ together with three peroxidases, NADPH-dependent thioredoxin reductase C (NTRC), monodehydroascorbate reductase 1 (MDAR1), and purple acid phosphatase (PAP26) (Table 2). Furthermore, among the 15 GO biological processes also appeared the term ‘response to oxidative stress’ containing 24 proteins (Supplementary Table S4), which included many of the above described and others such as chalcone synthase TT4 with a 7.8-fold change in persulfidation (Supplementary Table S2), MAP kinase 6, aconitase 3, glutathione peroxidase, thioredoxin M, and so on. Two glutathione-*S*-transferases were also present, including GSTF6, which showed a significant (*P*<0.05) change of 6.8-fold in persulfidation under drought (Supplementary Table S2), and among the glutathione-*S*-transferases, F12 showed the highest level of persulfidation, a 10.7-fold change, under drought conditions (Supplementary Table S2).

**Table 1.** Functional annotation by GO biological process of the proteins more persulfidated in plants under drought using Database for Annotation, Visualization, and Integrated Discovery (DAVID) bioinformatics resources (*P*<0.05)

Term	Count	Fold enrichment	FDR
Cellular response to oxidative stress	11	13.41	1.51 × 10^−6^
Hydrogen peroxide catabolic process	13	7.19	3.01 × 10^−5^
Response to cadmium ion	42	6.79	4.10 × 10^−19^
Carbohydrate metabolic process	33	4.30	3.91 × 10^−9^
Proteolysis	10	3.37	8.00 × 10^−2^
Protein folding	10	3.33	8.10 × 10^−2^
Response to cold	26	3.25	5.07 × 10^−5^
Response to heat	11	2.80	1.42 × 10^−1^
Response to oxidative stress	24	2.67	2.41 × 10^−3^
Cell wall organization	13	2.31	1.78 × 10^−1^
Small molecule metabolic process	10	2.29	3.62 × 10^−1^
Response to salt stress	23	2.20	3.16 × 10^−2^
Response to jasmonic acid	16	1.79	3.79 × 10^−1^
Response to abscisic acid	27	1.48	4.26 × 10^−1^
Response to light stimulus	30	1.45	4.17 × 10^−1^

**Table 2.** Persulfidated proteins classified within the GO biological processes ‘cellular response to oxidative stress’ and ‘hydrogen peroxide catabolic process’ in Arabidopsis plants under drought stress

Term	ID	Gene name
Cellular response to oxidative stress Hydrogen peroxide catabolic process	Q949U7	Thioredoxin superfamily protein (AT3G52960)
Cellular response to oxidative stress	Q9LU86	Thioredoxin superfamily protein (PRXQ)
Cellular response to oxidative stress Hydrogen peroxide catabolic process	Q05431	Ascorbate peroxidase 1 (APX1)
Cellular response to oxidative stress Hydrogen peroxide catabolic process	P82281	Ascorbate peroxidase 4 (TL29)
Cellular response to oxidative stress Hydrogen peroxide catabolic process	Q8GY91	Ascorbate peroxidase 6 (APX6)
Cellular response to oxidative stress	P24704	Copper/zinc superoxide dismutase 1 (CSD1)
Cellular response to oxidative stress	O78310	Copper/zinc superoxide dismutase 2 (CSD2)
Cellular response to oxidative stress	Q9LY14	Peptidemethionine sulfoxide reductase 3 (PMSR3)
Cellular response to oxidative stress Hydrogen peroxide catabolic process	Q42592	Stromal ascorbate peroxidase (SAPX)
Cellular response to oxidative stress Hydrogen peroxide catabolic process	Q9XEX2	Thioredoxin-dependent peroxidase 1 (TPX1)
Cellular response to oxidative stress Hydrogen peroxide catabolic process	Q42593	Thylakoidal ascorbate peroxidase (TAPX)
Hydrogen peroxide catabolic process	O22229	NADPH-thioredoxin reductase C (NTRC)
Hydrogen peroxide catabolic process	Q9SJZ2	Peroxidase superfamily protein (AT2G22420)
Hydrogen peroxide catabolic process	Q9SZB9	Peroxidase superfamily protein (AT4G33420)
Hydrogen peroxide catabolic process	Q9LFA3	Monodehydroascorbate reductase 1 (MDAR1)
Hydrogen peroxide catabolic process	Q9SMU8	Peroxidase CB (PRXCB)
Hydrogen peroxide catabolic process	Q949Y3	Purple acid phosphatase 26 (PAP26)

Several GO biological processes related to responses to abiotic stresses, such as cadmium ion, cold, heat, salt stress, and light stimulus, were also enriched under drought stress conditions, and contained more than 10 proteins each (Supplementary Table S5). It is worth highlighting the dehydration-associated proteins ERD10, RD29A, and ERD7, with 2.8-, 2.1-, and 1.4-fold higher levels of persulfidation, respectively. In addition, the protein phosphatase 2C HAB1 involved in the ABA signaling core (Hsu *et al.*, 2021) and also beta glucosidase 1 (BGLU1) showed a 2.9- and 18.5-fold change, respectively. The latter showed the second highest persulfidation change under drought and catalyses the hydrolysis of the glucose-conjugated biologically inactive ABA to the active form under dehydration conditions (Lee *et al.*, 2006). Interestingly, glyceraldehyde 3-phosphate dehydrogenase A and B, also described as targets of persulfidation (Aroca *et al.*, 2015), were included in these terms related to abiotic stresses. Furthermore, the GO enrichment analysis performed also showed terms related to response to abscisic acid and jasmonic acid (Supplementary Table S6), phytohormones involved in response and adaptation to drought stress (Kuromori *et al.*, 2022; other references within).

Two other terms, ‘proteolysis’ and ‘protein folding’, also showed more than 3-fold significant (*P*<0.05) enrichment (Table 1), and comprised different proteases, chaperones, and heat shock proteins (Supplementary Table S7).

Moreover, we also identified several GO molecular functions and Kyoto Encyclopedia of Genes and Genomes (KEGG) pathways with more than 3-fold enrichment, containing proteins with high persulfidation level under drought, which were connected with the GO biological process terms described above (Supplementary Table S8). Among them, some GOs linked to antioxidant activities were highlighted, as well as aminopeptidase activities and proteasome binding, and activities related to phytohormone metabolism. Interestingly, the term ‘3-mercaptopyruvate sulfurtransferase activity’ showed the highest level of fold enrichment (more than 45-fold change), which is in agreement with an increased protein persulfidation, since this activity is involved in the generation of persulfidated molecules (Selles *et al.*, 2019). Some of the enriched pathways of interest were anthocyanin (more than 22-fold change) and flavonoid (more than 5-fold change) biosynthesis, and glutathione and amino acid metabolism (Supplementary Table S8).

The proteins with lower persulfidation level in drought were analysed using the same criteria. The results showed 14 GO biological processes containing more than 10 proteins each (Supplementary Table S9). Curiously, those processes corresponded to the biosynthetic processes of glucosinolates and chlorophyll. Similarly, the analysis also showed that the GO molecular function term with the highest enrichment was ‘desulfoglucosinolate sulfotransferase activity’, and regarding the KEGG pathways, the highest enrichment corresponded to glucosinolate biosynthesis. In addition, the GO molecular function of ‘glutamate synthase activity’ was also included within the most enriched terms (Supplementary Table S10).

**Fig. 6.**
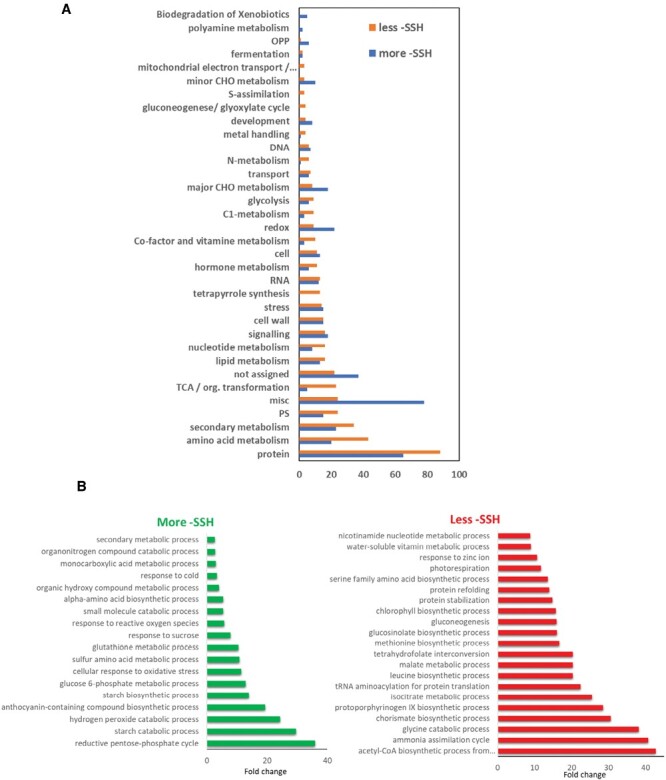
Functional classification of gene ontology (GO) terms of differentially persulfidated proteins in response to drought. (A) Classification categorized by biological processes according to MapMan. Bars are color coded as GO terms with proteins less persulfidated (-SSH) in orange and more persulfidated (-SSH) in blue. (B) GO enrichment performed using the PANTHER functional annotation tool for genes significantly more and less persulfidated (fold change >2, *P*<0.01) calculated with Fisher’s exact test and the Bonferroni correction applied for multiple testing.

Proteins differently persulfidated between drought and control conditions were also analysed using MapMan database (Fig. 6A). The classification revealed that the most abundant group corresponded to the protein bin considering both less and more persulfidated proteins in drought conditions. Furthermore, the subgroup involved in protein degradation was, in turn, the most abundant with 66 elements (39 proteins more persulfidated and 27 less persulfidated under drought conditions), including numerous proteases, several involved in UPS-dependent protein degradation (Supplementary Table S11), thus highlighting the importance of the alleviating effect of sulfide on protein-degradation pathways under drought conditions. In addition, a GO enrichment was performed using the PANTHER functional annotation tool for those proteins differentially persulfidated (Fig. 6B), and the results showed that within those proteins more persulfidated there was an enrichment of GO terms involved in abiotic stress response such as ‘hydrogen peroxide catabolic process’, ‘anthocyanin-containing compound biosynthetic process’, ‘cellular response to oxidative stress’, and ‘glutathione metabolic process’. Collectively, these results reinforce the conclusion that persulfidation regulates different processes to cope with the abiotic stress in response to drought.

## Discussion

H_2_S is now accepted as a regulatory/signaling molecule. It is equally as important as other signaling molecules in plant and animal systems, and depending on the concentration threshold shows a similar toxicity–signaling duality (Aroca *et al.*, 2020). In fact, exogenous application of sulfide donor molecules (such as NaHS used in this study) at low concentrations in the micromolar range has been used extensively in many plant studies and they show no toxicity symptoms, and only a relief effect in stressed plants has been described (Chen *et al*., 2011; Dooley *et al.*, 2013; Xie *et al.*, 2014; Li *et al.*, 2020b; Yao *et al.*, 2020). H_2_S is not only essential in regulating a wide range of vital processes, it also improves plant tolerance and protection against numerous adverse environmental conditions, particularly abiotic stress, allowing plant adaptability and resilience (Corpas, 2019; Gotor *et al.*, 2019; Aroca *et al.*, 2021b; Zhang *et al.*, 2021).

The data from this study further emphasize the role of H_2_S as a promoter of enhanced tolerance to environmental stress, not only because H_2_S pretreatment enables plants to respond more rapidly and efficiently after exposure to drought, but also because in the absence of exogenous H_2_S, a strong induction of endogenous H_2_S (Fig. 2) was observed in withstanding drought. Thus, plants pretreated with sulfide and subjected to drought exhibit phenotypic, biochemical, and molecular characteristics similar to plants grown under a non-stress situation, implying that drought stress conditions are alleviated by sulfide. Our findings support previous results demonstrating the role of H_2_S in improving drought resistance observed in different plant species either through exogenous application or by endogenous production (Jin *et al.*, 2011, 2018; Chen *et al*., 2016; Ma *et al.*, 2016; Zhou *et al.*, 2020; Amir *et al.*, 2021). In this study, a clear reduction in ROS content (Fig. 2) was observed in plants under drought when subjected to sulfide pretreatment, indicating that H_2_S contributes to balancing redox homeostasis. This agrees with several studies showing that H_2_S allows plants to adapt to adverse conditions through increased antioxidative defenses (Gotor *et al.*, 2019). In addition, the proteomic analysis performed in this work provides further information about the mechanism of endogenous H_2_S in combating drought-induced oxidative stress. Although several mechanisms of action have been proposed, our findings suggest a main role of protein persulfidation, and this has been well-established in recent years (Aroca *et al.*, 2018, 2021b; Gotor *et al.*, 2019, 2022). Our analysis revealed that the most enriched groups containing the most persulfidated proteins in drought (Table 1) are involved in cellular response to oxidative stress and the hydrogen peroxide catabolic process, both containing numerous antioxidative enzymes (Table 2). This is in agreement with the persulfidation of Arabidopsis proteins, with APX1 at Cys32 (Aroca *et al.*, 2015), and tomato proteins, with cytosolic ascorbate peroxidase 1 at Cys168, catalase 1 at Cys234, and peroxidase 5 at Cys46 and Cys61, which increases the activities of these enzymes and enhances resistance to oxidative stress (Li *et al.*, 2020a).

In addition, several proteins involved in response to a diversity of abiotic stresses are also included in the list of enriched groups under drought (Table 1), confirming the extensive existing data on the role of H_2_S in plant adaptation to adverse conditions and suggesting that the most feasible mechanism of action is protein persulfidation (Aroca *et al.*, 2021b). Besides, enrichment analysis also showed processes related to response to jasmonic and abscisic acids (Table 1), plant hormones well-known as components of the stress signaling cascade (Waadt *et al.*, 2022). ABA plays critical roles mediating drought stress responses by regulating stomatal closure and stress-responsive gene expression through the activation of a complex signaling pathway (Cutler *et al.*, 2010). The interconnections between H_2_S and ABA have been extensively described in plant responses to different environmental stresses, and an important insight into the H_2_S action in ABA-dependent stomatal closure has recently been revealed (Aroca *et al.*, 2021b; Pantaleno *et al.*, 2021; Siodmak and Hirt, 2021). In guard cells, a complex crosstalk between the H_2_S and ABA signaling networks has been demonstrated, which occurs through the persulfidation of specific proteins (Shen *et al.*, 2020; Chen *et al.*, 2021; Zhou *et al.*, 2021). Overall, this study also contributes to deciphering how H_2_S promotes enhanced tolerance/resilience to drought, describing numerous targets that undergo persulfidation to regulate a wide range of processes involved in response to drought.

To cope with abiotic stress conditions, plants activate different strategies to maintain growth and ensure survival, including adaptations in amino acid metabolism (Heinemann and Hildebrandt, 2021). Our study shows that the content of some amino acids (Fig. 3) was unchanged, while others, such as aromatic amino acids, branched-chain amino acids, and Met and Thr accumulated substantially under drought stress. These findings are consistent with the conclusion that this accumulation is a consequence of increased protein turnover during abiotic stress, but also of the synthesis of specific amino acids as precursors of secondary metabolites involved in plant tolerance (Hildebrandt, 2018; Batista-Silva *et al.*, 2019). In this way, the accumulation of the aromatic amino acids Tyr and Phe under drought stress could be due to the biosynthesis of different flavonoids identified in Arabidopsis, including anthocyanins (Saito *et al.*, 2013). In addition, Met, Trp, and Phe are precursors of glucosinolates that have been shown to be involved in abiotic stress resistance (Salehin *et al.*, 2019). In accordance with this, our proteomic studies show that biosynthesis of anthocyanin, flavonoid, and glucosinolates is among the enriched pathways regulated by persulfidation (Fig. 6).

The UPS and autophagy are two complementary protein degradation pathways responsible for homeostasis of cellular components following various internal and external cues. The UPS and autophagy are linked in many ways, such as ATG8 protein being lipidated by ubiquitin-like processes, ubiquitin being involved in some selective autophagy processes, the components of the UPS being degraded by autophagy, and ATGs being degraded by the UPS (Su *et al.*, 2020; Clavel and Dagdas, 2021). Both pathways are essential to regulate plant responses to drought, which is reinforced by our findings not only at the biochemical level but also from the proteomic analyses. Interestingly, our results demonstrated that H_2_S negatively regulates autophagy (Fig. 4) and protein ubiquitination (Fig. 5) induced by drought in a similar way as previously demonstrated when autophagy is induced by other situations (Aroca and Gotor, 2022).

In conclusion, our data suggest a role of H_2_S as a promoter of enhanced tolerance to drought stress through protein persulfidation. Comparative and quantitative analyses reveal that the major group of persulfidated proteins are involved in combating the oxidative stress caused by drought conditions. Alleviation by H_2_S-dependent persulfidation also involves adaptations in amino acid metabolism and regulation of protein degradation pathways.

## Supplementary data

The following supplementary data are available at *JXB* online.

Table S1. Oligonucleotides used in this study for real-time RT-PCR analysis.

Table S2. Identified proteins more persulfidated in plants under drought stress.

Table S3. Identified proteins more persulfidated under control conditions.

Table S4. Proteins classified within the GO biological process ‘response to oxidative stress’ in plants under drought stress.

Table S5. Proteins classified within the GO biological process ‘response to different abiotic stresses’ in plants under drought stress.

Table S6. Proteins classified within the GO biological process ‘responses to hormones’ in plants under drought stress.

Table S7. Proteins classified within the GO biological processes ‘proteolysis’ and ‘protein folding’ in plants under drought stress.

Table S8. Functional annotation by GO molecular function and KEGG pathway with more than 3-fold significant enrichment in plants under drought stress.

Table S9. Functional annotation by GO biological process of the proteins less persulfidated in plants under drought stress.

Table S10. Functional annotation by GO molecular function and KEGG pathway with more than 3-fold significant enrichment in Arabidopsis plants under control conditions.

Table S11. Persulfidated proteins identified involved in protein degradation using MapMan database.

Dataset S1. Persulfidated proteins identified in all samples.

Dataset S2. Differentially persulfidated proteins in response to drought stress.

erad165_suppl_Supplementary_Datasets_S1-S2

erad165_suppl_Supplementary_Table_S1

erad165_suppl_Supplementary_Tables_S2-S11

## Data Availability

The mass spectrometry proteomics data have been deposited in the ProteomeXchange Consortium via the PRIDE partner repository with the dataset identifiers PXD039999.
