# Supplementary material for: Sulfide promotes tolerance to drought through protein persulfidation in Arabidopsis
Source: J Exp Bot. 2023 May 6;74(15):4654–69. doi: 10.1093/jxb/erad165 (PMC10433926; doi:10.1093/jxb/erad165)
Supplement: erad165_suppl_Supplementary_Table_S1 [file erad165_suppl_supplementary_table_s1.pdf]

Table S1. Oligonucleotides used in this study for real-time RT-PCR analysis.

Oligonucleotides	Sequence
RD29A-F	AAATTGTTCGGCTTTGGATT
RD29A-R	CAACACCTCAACAAGTCACACT
RAB18-F	GAACATGGCGTCTTACCAGA
RAB18-R	ATCGGATTTCCGTACTCGTC
ATG8a-F	CGATCTTTGGATGACTTTGATG
ATG8a-R	TGACGATTAATAAACCCAAAGG
ATG8b-F	TTGGCCAATTTGTGTACGTT
ATG8b-R	TCCACCAAATGTGTTCTCTCC
ATG8c-F	TGAGTGCCGAAAAGGCTATC
ATG8c-R	ACCAAACCAAAGGTGTTCTCT
ATG8d-F	TTTGACTGTTGGCCAGTTTG
ATG8d-R	AACCCGTCTTCGTCTTTGTG
ATG8e-F	CTGACCTCTGAGAATCCGCC
ATG8e-R	AGCCACACAAACTTATAATACCAAA
ATG8f-F	GCTGGATATTTCATTTTACTCCTC
ATG8f-R	GAGGGAGATGTGATAGATTCA
ATG8g-F	TGTGATTCGTAAGAGAATCCAAC
ATG8g-R	CCAAAAGTGTTTTCCCCACT
ATG8h-F	CCAAAGCTCTCTTTGTTTTCG
ATG8h-R	AAGAACCCGTCTTCTTCCTTG
ATG8i-F	TGTCAACAACACTCTCCCTCA
ATG8i-R	AACCAAAGGTTTTCTCACTGC
